# Development and Evaluation of a Pedagogical Tool to Improve Understanding of a Quality Checklist: A Randomised Controlled Trial

**DOI:** 10.1371/journal.pctr.0020022

**Published:** 2007-05-04

**Authors:** Lola Fourcade, Isabelle Boutron, David Moher, Lucie Ronceray, Gabriel Baron, Philippe Ravaud

**Affiliations:** 1 Université Paris 7 Denis Diderot, UFR de Médecine, Paris, France; 2 Assistance Publique-Hôpitaux de Paris (AP-HP), Hôpital Bichat, Département d'Epidémiologie, Biostatistique et Recherche Clinique, Paris, France; 3 INSERM, U738, Paris, France; 4 Children's Hospital of Eastern Ontario Research Institute, University of Ottawa, Canada; 5 Department of Pediatrics, Faculty of Medicine, University of Ottawa, Canada

## Abstract

**Objective:**

The aim of this study was to develop and evaluate a pedagogical tool to enhance the understanding of a checklist that evaluates reports of nonpharmacological trials (CLEAR NPT).

**Design:**

Paired randomised controlled trial.

**Participants:**

Clinicians and systematic reviewers.

**Interventions:**

We developed an Internet-based computer learning system (ICLS). This pedagogical tool used many examples from published randomised controlled trials to demonstrate the main coding difficulties encountered when using this checklist.

Randomised participants received either a specific Web-based training with the ICLS (intervention group) or no specific training.

**Outcome measures:**

The primary outcome was the rate of correct answers compared to a criterion standard for coding a report of randomised controlled trials with the CLEAR NPT.

**Results:**

Between April and June 2006, 78 participants were randomly assigned to receive training with the ICLS (39) or no training (39). Participants trained by the ICLS did not differ from the control group in performance on the CLEAR NPT. The mean paired difference and corresponding 95% confidence interval was 0.5 (−5.1 to 6.1). The rate of correct answers did not differ between the two groups regardless of the CLEAR NPT item. Combining both groups, the rate of correct answers was high or items related to allocation sequence (79.5%), description of the intervention (82.0%), blinding of patients (79.5%), and follow-up schedule (83.3%). The rate of correct answers was low for items related to allocation concealment (46.1%), co-interventions (30.3%), blinding of outcome assessors (53.8%), specific measures to avoid ascertainment bias (28.6%), and intention-to-treat analysis (60.2%).

**Conclusions:**

Although we showed no difference in effect between the intervention and control groups, our results highlight the gap in knowledge and urgency for education on important aspects of trial conduct.

## Editorial Commentary

**Background:** A key part of the practice of evidence-based medicine (essentially, the appropriate use of current best evidence in determining care of individual patients) involves appraising the quality of individual research papers. This process helps an individual to understand what has been done in a clinical research study, and to decipher the strengths, limitations, and importance of the work. Several tools already exist to help clinicians and researchers to assess the quality of particular types of study, including randomised controlled trials. One of these tools is called CLEAR NPT, which consists of a checklist that helps individuals to evaluate reports of nonpharmacological trials (i.e., trials not evaluating drugs but other types of intervention, such as surgery). The researchers who developed CLEAR NPT also produced an Internet-based computer learning system to help researchers use CLEAR NPT correctly. They wanted to evaluate to what extent this learning system helped people use CLEAR NPT and, therefore, carried out a randomised trial comparing the learning system to no specific training. A total of 78 health researchers were recruited as the “participants” in the trial, and 39 were randomised to each trial arm. Once the participants had received either the Internet training or no specific training, they used CLEAR NPT to evaluate reports of nonpharmacological trials. The primary outcome was the rate of “correct” answers that study participants gave using CLEAR NPT.

**What the trial shows:** The researchers found that the results on the primary outcome (rate of correct answers given by study participants) did not differ between the study arms. The rate of correct answers for individual items on the checklist also did not seem to differ between individuals receiving Internet training and those receiving no specific training. When looking at the scores for individual items, combined between the two study arms, participants scored highly on their appraisal of some aspects of trial design (such as generation of randomisation sequences and descriptions of blinding and the intervention) but poorly on other items (such as concealment of the randomisation sequence).

**Strengths and limitations:** Key strengths of this study include the randomised design and that the trial recruited enough participants to test the primary hypothesis. The failure to find a significant difference between study arms in this trial was likely not due to a lack of statistical power. One limitation of the study is that the group of researchers who participated were already fairly experienced in assessing trial quality at the start, and this may explain why no additional effect of the computer-based learning system was seen. It is possible that the training system may have some benefit for individuals who are less experienced in evaluating trials. A further possible limitation may be that there was a small imbalance at randomisation, with slightly more experienced researchers being recruited into the arm receiving no specific training. This imbalance might have underestimated the effect of the training system.

**Contribution to the evidence:** The researchers here report that this study is the first they are aware of that evaluates a computer-based learning system for improving assessment of the quality of reporting of randomised trials. The results here find that this particular tool did not improve assessment. However, the results emphasise that training should be considered an important part of the development of any critical appraisal tools.

## INTRODUCTION

Assessing the quality of reports of randomised controlled trials (RCTs) is particularly important for clinicians' critical appraisal of the health-care literature and for systematic reviewers [1]. In fact, evidence suggests that inadequate reporting is associated with biased treatment effect estimates [2–5]. The QUOROM (Quality of Reporting of Meta-analysis) Statement [6] recommends reporting the criteria and the process used for quality assessment of trials included in a systematic review or meta-analysis. Similar recommendations can also be found in section 6 of the Cochrane Handbook for Systematic Reviews of Interventions [7]. Moja et al. recently reported that the methodological quality of primary studies was assessed in 854 of 965 systematic reviews (88.5%) [8].

Quality assessment is often achieved by the use of checklists or scales, such as the Veerhagen list or the Jadad scale [9–12]. In the field of nonpharmacological treatment (NPT), a checklist—the checklist to evaluate a report of a nonpharmacological trial (CLEAR NPT)—was developed to assess the quality of RCTs included in meta-analysis [13]. This assessment tool was developed using the Delphi Consensus method, with consensus of 55 international experts (clinicians, methodologists, and members of the Cochrane collaboration). It includes ten items and five subitems (Text S1) and is published with a user's guide explaining each item in detail (Text S2).

Reproducibility issues have been raised regardless of the chosen quality tool [14], because inconsistently defined items such as blinding [15], dropout and withdrawals [16], or an intention-to-treat analysis [17–20] are used and are poorly understood by reviewers. To overcome these issues, some authors have developed specific guidelines for some quality tools, which provide detailed explanation on scoring each item [9]. Further, a training session is recommended for all reviewers [9]. Despite these recommendations, Clark et al. showed that in a study of reviewers with face-to-face training sessions before scoring reports of RCTs, the interrater agreement for the Jadad scale— one of the simplest quality tools—was poor (kappa 0.37 to 0.39) [16]. Therefore, other pedagogical tools to improve the understanding and the reproducibility of these scales and checklists are needed.

### Objectives

To improve the understanding of the CLEAR NPT, we developed an Internet-based computer learning system (ICLS). This pedagogical tool offers, through the use of practical examples from RCTs, a problem-based approach to solving the main coding difficulties encountered when using the CLEAR NPT. We chose a Web-based tool as it is more feasible than face-to-face meetings and can be tailored to individuals' answers. To evaluate the impact of the ICLS on proper coding with the CLEAR NPT, we carried out an RCT comparing ICLS to no specific training.

## METHODS

### Development of ICLS

The ICLS was developed in three steps: construction, design, and validation.

#### Construction of the ICLS database.

To develop the ICLS, we identified difficulties encountered when using the CLEAR NPT (e.g., lack of comprehension of the items and lack of consistency in the definition of an item) and selected passages from RCTs that could be include in the ICLS.

For this purpose, we selected a panel of reports of RCTs assessing NPT (Text S3).

Two reviewers, one involved in the elaboration of the CLEAR NPT (IB) and one using the CLEAR NPT for the first time (LF), independently assessed these reports using the CLEAR NPT items. A meeting followed in which the ratings were compared. This session allowed for the identification of disagreement and difficulties in understanding CLEAR NPT items. According to the difficulties in understanding CLEAR NPT items for this panel, the two reviewers selected specific passages that were either adequately reported, inadequately reported, or a frequent cause for disagreement.

Although reviewers can be non-native English speakers, the computer learning system was written in English. In fact, most papers included in systematic reviews and meta-analyses are published in English. Consequently, it seemed logical to use the same language in the learning system.

#### Designing the ICLS program.

We designed a computer program following the model of a knowledge-based expert system [21–23]. The main principles of this program are reported in Figure S1. After proposing a short passage from a clinical trial previously selected for the database, the first item is put forward for participants with its modalities of answers (e.g., yes/no/unclear). Depending on their answers, users are led on different pathways drawn from the CLEAR NPT user's guide: (1) If the answer is correct, users are directed to a Web page confirming the correct answer for this item, which also provides a detailed explanation and computerized version of the user's guide; (2) If the answer is incorrect, participants are asked a list of subquestions to help them determine where they made a mistake. The system is therefore self-correcting and enhances understanding of incorrect participant answers. Each participant has a minimum of two passages to refer to for each item and one last passage if they answered incorrectly for their second passage.

#### Validation of the ICLS.

The computer learning system was validated by one of the authors (PR) who confirmed the validity of the answers and pathways of the ICLS. The ICLS was also tested by a group of three people who had never used CLEAR NPT.

### The RCT: Influence of the ICLS on Coding with the CLEAR NPT

We designed an RCT comparing two groups of participants receiving either the user's guide and specific training with the ICLS (intervention group) or a user's guide with no specific training (control group) to assess the impact of the ICLS. In France, the submission of a trial to an ethics committee is defined according to the public health law of August 2004, which requires the submission of protocols for review by an ethics committee only if the trial involves patients, and if the treatment is not administered in clinical practice but involves a specific treatment or specific investigation. Trials aimed at educating medical doctors or reviewers are not required to submit the protocol to an ethics committee. Participants in our study were previously informed of the trial, they could withdraw from the trial if they wished, and they were informed of the results of the trial upon completion.

#### Participants.

Members from three different categories of participants were invited by e-mail to participate in the RCT: (1) Members of Health Technology Assessment international (HTAi) (*n* = 430) were selected for their knowledge of quality assessment. HTAi is an international society for the promotion of health technology assessment and holds international conferences and forums. Members are involved in the field of evaluation, and some perform systematic reviews; (2) directors of Evidence-based Practice Centers (EPC) (*n* = 13) who develop systematic reviews and technology assessments on topics relevant to clinical, social science/behavioral, economic, and other healthcare organization and delivery issues; and (3) corresponding authors of meta-analyses of NPT published between 1 January 2004 and 3 March 3 2006 (*n* = 100).

#### Design.

Participants were randomised in pairs to be evaluated at the end on the same report (i.e., each report was evaluated with CLEAR NPT by one participant in both groups). This design allowed for assessing reviewers' understanding of several articles. A smaller panel would decrease the variability of the results. However, the quality of reporting of the trial is a critical issue in quality assessment, and we would not have been able to formulate conclusions on the basis of a smaller panel.

#### Randomisation: Sequence generation.

The paired randomisation procedure was centralized and performed by means of a computer-generated list stratified on the degree of expertise in the field of meta-analysis by a statistician of the epidemiology department performing the trial.

#### Randomisation: Allocation concealment.

The investigators did not have access to this procedure. Participants were considered “experts” if they had been involved in the publication of a meta-analysis indexed in PubMed.

#### Randomisation: Implementation.

The randomisation was implemented on the Web site by a computer scientist (LR). Participants could not foresee their assignment until the beginning of the intervention. They received a personal log-in account number by e-mail that directed them to an appropriate Web page depending on their randomisation group. Each pair of participants assessed one report of a randomised trial. A waiting list of participants who agreed to be randomised was compiled to replace withdrawals.

#### Interventions.

Participants in both groups received an e-mail containing the CLEAR NPT checklist and the user's guide. For each item in the CLEAR NPT, the user's guide explained its meaning and how to score it. The checklist and user's guide are detailed in Texts S1 and S2.

The control group received only the user's guide and was asked to assess one report of a randomised trial using the CLEAR NPT, whereas the experimental group was directed to the ICLS and also assessed one report of a randomised trial after completing the training.

#### Blinding (masking).

Participants could not be blinded to their randomisation group because of obvious differences in terms of intervention.

#### Panel of reports assessed by the participants.

To evaluate the performance of participants in using CLEAR NPT, we selected a panel of RCTs by searching PubMed for all RCTs assessing NPTs published between 1 January 2005 and 31 March 2006, in the following journals: *New England Journal of Medicine,* the *Journal of the American Medical Association, Lancet, Annals of Internal Medicine, BMJ, Annals of Surgery, British Journal of Surgery, Annals of Surgical Oncology, Archives of General Psychiatry, American Journal of Psychiatry, Journal of Clinical Psychiatry, Physical Therapy, Supportive Care in Cancer,* and *Archives of Physical Medicine and Rehabilitation.*

A total of 200 reports were identified. Among these, some reports were randomly selected to be evaluated. Half of these reports assessed a surgical procedure, and half assessed another NPT such as rehabilitation, psychotherapy, or devices. The selected articles are described in Text S4.

#### Outcomes.

Three reviewers (LF, IB, and PR) independently assessed the selected reports. All discrepancies were discussed, and the user's guide was consulted to obtain a consensus for appropriate answers for each item of the CLEAR NPT. This consensus was considered as the criterion standard.

At the end of the training program, participants had to assess one of the selected reports of an RCT using the CLEAR NPT and complete a qualitative assessment of the ICLS. The primary outcome was the rate of correct answers on the ten main items of each group for the final assessment compared to the criterion standard. Secondary outcomes were the rate of correct answers for each item and a qualitative assessment of the ICLS by the survey participants, completed after fulfilling the training program.

#### Sample size.

A sample size of 38 pairs will have 85% statistical power to detect a difference in means of 10% (e.g., a mean rate of correct responses of 70% in the intervention group and 60% in the control group), assuming a standard deviation of differences of 20%, using a paired Student's t-test with a 0.05 two-sided significance level.

#### Statistical methods.

The mean rate of correct answers of participants to the criterion standard was compared by a paired Student's t-test. The “per item rate” of correct answers to the criterion standard was compared by use of a McNemar test for paired dichotomous data and with Yates correction when appropriate. A *p*-value ≤ 0.05 was considered statistically significant, and all tests were two-sided. Statistical analyses involved the use of SAS 9.1 (SAS Institute, http://www.sas.com).

## Results

### Participant Flow

**Figure 1:**
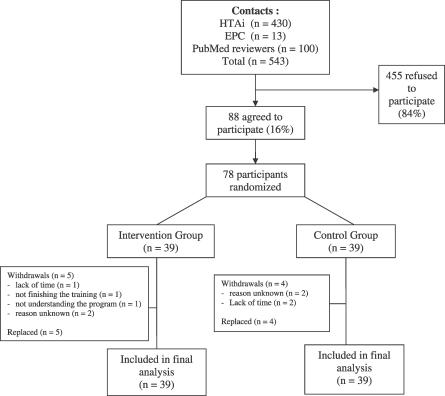
Flow Chart of Participants

Figure 1 shows the flow of participants through the trial. Of the 543 people invited to participate, 88 agreed to participate (16%), and 78 were randomised, 39 allocated to receive training with the ICLS and 39 to receive no training. A total of nine participants did not complete the survey and were replaced by waiting list participants. The main reasons for withdrawals were not having time to complete the survey (i.e., spontaneous withdrawal, *n* = 3), not understanding the program (*n* = 1), not completing the survey after five reminders (*n* = 4), and not finishing the training (*n* = 1).

### Numbers Analysed

A total of 78 participants completed the final assessment and were analysed.

### Recruitment

Between April and June 2006, 78 participants were recruited

### Baseline Data

Baseline characteristics are described in Table 1. The number of meta-analyses published on PubMed was similar in each group. However, despite stratifying on expertise with meta-analysis, the declared expertise was higher in the control group (84.2%) than in the intervention group (65.8%) (Table 1). Among the participants four, had already used the CLEAR NPT. The description of the ICLS and panel of articles used for final assessment is described in Text S5.

**Table 1:**
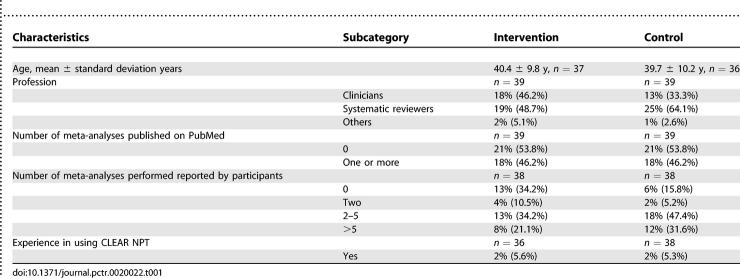
Baseline Characteristics of Participants

### Outcomes and Estimation

#### Primary outcome.

**Figure 2:**
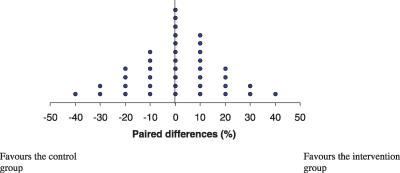
Dot Plot of the 39 Paired Differences Each plot represents a paired difference (i.e., the rate of correct responses of the intervention respondent minus the rate of correct responses of the control respondent). Observation to the left of 0 favours the control group and observation to the right of 0 favours the intervention group.

Results on the primary outcome results are reported in Figure 2. The performance of participants trained by the ICLS did not differ from that of the control group. The mean paired difference and corresponding 95% confidence interval was 0.5 (−5.1 to 6.1).

#### Secondary outcomes.

Regardless of the CLEAR NPT checklist item considered, the rate of correct answers did not differ between the two groups (Table 2). Overall, taking into consideration all participants, the rate of correct answers was high for the items related to the allocation sequence (79.5%), the description of the intervention (82.0%), blinding of patients (79.5%), and follow-up schedule (83.3%). The rate of correct answers was low for items related to the allocation concealment (46.1%), co-interventions (30.3%), blinding of outcome assessors (53.8%), specific measures to avoid ascertainment bias (28.6%) and intention-to-treat analysis (60.2%).

**Table 2:**
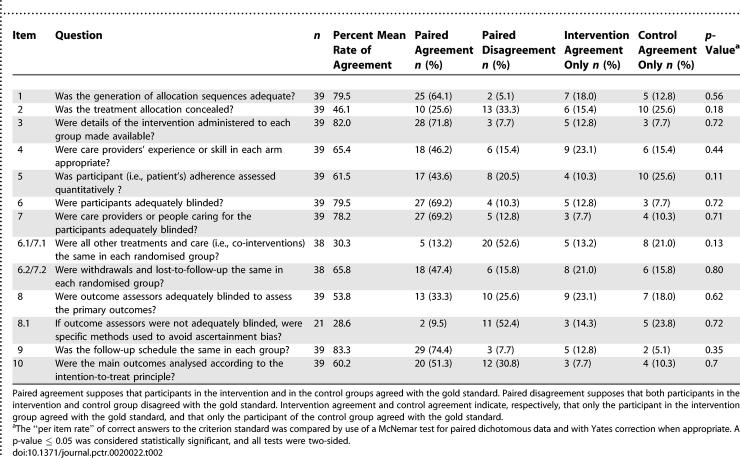
Rate of Correct Answers Per Group Per Item

## DISCUSSION

### Interpretation

To our knowledge this study is the first to develop and evaluate a computer learning system to improve the understanding of a checklist for assessing the quality of reporting of RCTs. Moher et al. reported an annotated bibliography of scales and checklists developed to assess quality [9]. Only a few quality tools have clear users' guides to standardize the understanding of the items, and none are provided with a specific training program. This computer learning system is Internet-based so it offers greater flexibility in training time and sequencing. We assessed the impact of the ICLS in assessing reports of RCTs of NPTs. Although participants were satisfied with the quality of the computer program (interface, readability of the text, and information delivered), training with the ICLS did not have a significant and relevant impact in terms of rate of correct answers compared with a criterion standard. These results highlight the difficulties in training and are consistent with systematic reviews showing that for peer review, referees' training did not improve the quality of the review [24,25]. However, in this trial we cannot determine whether the problem was related to the quality instrument or to the teaching tool.

### Overall Evidence

Some factors can be offered to explain the lack of efficacy of the ICLS. First, most of the participants had been involved in the publication of at least one meta-analysis. Consequently, this population has some level of expertise in quality assessment and probably needs more specific training than naïve participants. Consequently, we should probably assess the impact of the ICLS on inexperienced participants to determine its effect on the performance of this population.

Second, the ICLS trained participants similarly for each item of the checklist. However, our results highlighted that lack of reproducibility concerned only some items of the checklist. Items related to the allocation sequence generation, description of interventions, blinding of patients or healthcare providers, and follow-up schedule were well rated, with more than 80% correct answers. These items probably need little or no training for proper scoring. Consequently, the ICLS could be tailored and provide more training on various examples for items with low understanding.

Third, some examples of RCTs used to question and train a reviewer when using the CLEAR NPT might not be adequate. In fact, some examples with a high rate of correct answers were probably less informative, whereas other examples with a low rate of correct answers were probably more valuable for educating reviewers.

Finally, because participants were not blinded to the aim of the study, we cannot exclude the risk of bias with participants in the control group relying on the user's guide with more attention than they would do in usual practice.

Although the ICLS had no significant impact on RCT reviewers' performance, most assessment tools do not have any instructions in how to use the quality assessment scale [9–12], whereas the training can be viewed as proactive and is recommended as an important component in the presentation of any new instrument development. Our results highlighted the lack of consistent understanding of some items. These results could be linked to: (1) the wording of the items of the checklist that could be slightly modified; (2) a lack of consensus on the definition of some items; and (3) an inadequate reporting of the trial that could have been confusing for reviewers. Lack of adequate understanding concerned items specific to the CLEAR NPT such as co-interventions, specific methods to avoid ascertainment bias, and participant adherence but also items assessed in most quality tools such as allocation concealment, intention-to-treat analysis, and blinding of outcome assessors, which are key weapons in the fight against bias. For example, a debate arose when considering the results of the Balk et al. series, because the authors considered that an opaque sealed envelope was an adequate method of allocation concealment [4,26]. These results need to be highlighted, considering the high degree of expertise our participants have in the field of peer review and point out the need for education on these topics among the scientific community.

The reproducibility of the items specific to the CLEAR NPT could probably be improved upon with a modification of the wording of these items. The item “Was participants adherence assessed quantitatively?” could be clarified with the following wording “Was participants adherence reported quantitatively in the results section?”. Furthermore, the item on co-interventions, which requires that the description of the co-intervention be provided in the results section not only in the methods section, could be modified as follows: “Were all other treatments or care as described in the results section the same in each randomised group?”

Our results show that the item “Was the treatment allocation concealed?” had fewer than 50% of correct answers. These results are probably linked to the lack of consistency of the definition of allocation concealment. Pildal et al. [27] recognized that, depending on the reviewer, strict or loose criteria could be used to define allocation concealment. According to the definition used, sealed envelopes not reported as opaque would be considered as an adequate or inadequate method of concealment. In our study, we defined allocation concealment according to strict criteria, as Schulz et al. related regarding deciphering the allocation sequence by taking the nonopaque sealed envelopes to a “hot light” [28]. Some reviewers require an even more strict definition of allocation concealment with the need to report who prepared the envelopes. In fact, if the same person prepared the envelopes and recruited the patients, the allocation would not be adequately concealed.

Blinding of the outcome assessors was also poorly rated. These results concerned mainly trials in which the main outcome was a patient-reported outcome but patients were not reported as blinded. Therefore, the outcome assessor (i.e., the patient) could not be considered as adequately blinded even if the authors mentioned the presence of a blinded data collector who questioned the patients.

Only 60% of participants were in agreement with the criterion standard for the item related to intention-to-treat analysis. These results are probably linked to the poor reporting of this issue [17,29]. Baron et al. [30] showed that, in a panel of 81 reports, 66.7% described an intention-to-treat analysis, but full intention to treat was performed in only 7.4% of the studies.

These results are consistent with other studies. Maher et al. [31] evaluated the reliability of the ten-item Physiotherapy Evidence-Based Database (PEDro) scales and found kappa scores ranging from 0.12 to 0.73 (0.36 to 0.80 for individual assessors) for items, with low concordance on intention-to-treat analysis and therapist blinding. Clark et al.[16] showed a poor interrater agreement (kappa score range 0.37–0.39) for the Jadad scale.

### Limitations

The validity of the criterion standard used to assess raters' performance when using the CLEAR NPT could be a limitation. However, this standard was developed by three reviewers, two of whom were involved in the elaboration of the CLEAR NPT. They evaluated all 39 reports independently and according to the user's guide. They discussed all the discrepancies to come to a consensus.

### Generalisability

Another limitation is related to the rate of participation: 84% of reviewers approached did not participate. The time necessary to participate in this trial could likely explain these results. This may limit the generalisability of the results.

Finally, the baseline distribution of reviewers was imbalanced, with more experienced meta-analysts randomised to the control group. The effect of this potential baseline imbalance could dilute the intervention effect.

In conclusion, in this study, we attempted to improve the understanding of a quality checklist that evaluates reports of nonpharmacological trials, the CLEAR NPT, with an ICLS. Although this pedagogical tool did not improve participants' performance in using the checklist, our results highlight the lack of consistent understanding of some of the key weapons in the fight against bias. There is an urgent need for specific training to improve the understanding of such quality tools.

## SUPPORTING INFORMATION

CONSORT Checklist (48 KB DOC)

Trial Protocol Original protocol (84 KB DOC)

Trial Protocol Amended protocol (28 KB DOC)

Figure S1 Main Principles of the ICLS (43 KB DOC)

Text S1 CLEAR NPT (43 KB DOC)

Text S2 User's Guide for the Checklist of Items Assessing the Quality of Randomised Controlled Trials of NPT (82 KB DOC)

Text S3 Panel of Reports Used to Develop the Computer Learning System. (50 KB DOC)

Text S4 Panel of Reports Used for the Final Assessment of Participants (51 KB DOC)

Text S5 Description of the ICLS and Panel of Articles Used for Final Assessment (102 KB DOC)

## Abbreviations

CLEAR NPT: checklist to evaluate a report of a nonpharmacological trial
ICLS: Internet-based computer learning system
NPT: nonpharmacological treatment
RCT: randomised controlled trial
