# Supplementary material for: Development and Evaluation of a Pedagogical Tool to Improve Understanding of a Quality Checklist: A Randomised Controlled Trial
Source: PLoS Clin Trials. 2007 May 4;2(5):e22. doi: 10.1371/journal.pctr.0020022 (PMC1865084; doi:10.1371/journal.pctr.0020022)
Supplement: Trial Protocol — (84 KB DOC) [file pctr.0020022.sd002.doc]

Does a computer learning system improve the understanding and dissemination of CLEAR NPT?

## Protocol

# Lola Fourcade, Isabelle Boutron, David Moher, Peter Tugwell, Philippe Ravaud

1. **Background**

In order to evaluate the quality (internal validity) of randomized controlled trials assessing NPT ^1, 2^(e.g., surgery, technical operations, angioplasty, joint lavage, use of technical devices, radiotherapy, transcutaneous electrical nerve stimulation, rehabilitation, behavioural therapy, natural health practices (yoga), and psychotherapy) a checklist of items was developed using the Delphi Consensus Method.^3, 4^

This checklist, called CLEAR NPT, includes 10 items and 5 sub-items and is presented with a “users’ guide” explaining in detail the meaning of each item.

1. **Objective**

To develop a computer learning system (CLS) to train physicians and/or systematic reviewers to use the CLEAR NPT checklist.

1. **Method**

α) “Review”

*Method: We reviewed the literature about CLS used for personal and didactic training in:

-Pub Med using the words “computer assisted instruction”, “computer assisted learning system”, “Integrated learning system” ^5-8^

-The Cochrane library using the same words as used previously

-ERIC (educational resources information center)

-Psyclit

-Embase (excerpta medica)

-Yahoo: searching for “computer assisted instruction”, “computer assisted learning”,

“knowledge based expert system”

*Result: we selected “20” papers^5-25^ that showed the type of CLS system used in medicine and other fields and the common process of assessing their validity and reliability.

β) Practicing CLEAR NPT

We selected a number of 40 reports (randomized controlled trials using non pharmacological treatments).

A group of 2 people (IB, LF) [One involved in the elaboration of CLEAR NPT, one using it for the first time] assessed the reports using the CLEAR NPT items.

Then we listed difficulties encountered during this process (lack of comprehension of the items, lack of data in the text, main issues to understand the question proposed by each items…etc…).

Regarding these difficulties, we selected text from these reports that a reviewer would question when using CLEAR NPT; these passages became the database of the computer program.

γ) Designing the program

We designed a computer program following the model of a knowledge-based expert system.

We first entered the database in the program.

We then designed for each item a chain of possible answers and consequences based on this model:

The first item is proposed to participants (with an adequate sample of reports)

Wrong answer Right answer Explanation of the answer

based on the “user’s guide”

previously given to the participants

A sub-item is to enhance memorization of the item

proposed to respondents and its possible answers.

to improve the understanding

of the previous item

This sub-item can be a list of Second item is proposed

questions giving details about

the first item (i.e., which question

should one ask when reading

the first item).

Right answer to Wrong answer to the sub-item

the sub-item

User guide slide Another Explanation of the answers of items

explanation of the sub-item is AND sub-items based on the “user’s

correct answer for designed guide”

this example, then

go to the next item

Explanation for this item

User guide slide

Then go to the next item

This program should enhance the understanding and the proper use of the CLEAR NPT by stimulating the reflexion of participants who can understand their own mistakes and correct them spontaneously.

δ) Evaluation

**Study design**: Randomised controlled trial comparing two groups of participants:

-intervention group: trained with the computer learning system,

-control group: no specific training

**Participants**: All the Cochrane Review Groups will be invited to participate. Reviewers who published a Cochrane review will also be invited to participate in this trial.

The randomisation will be centralized in the Department of Clinical Research at Hospital Bichat.

Reviewers agreeing to participate will be randomised according to a computer-generated randomisation list. Reviewers randomised in the intervention group will receive an e-mail with an internet link to the CLEAR NPT and its user’s guide and a log-in for the computer learning system. Training takes between 30 to 45 minutes.

The control group will receive a link to the CLEAR NPT and its user’s guide.

All participants will have to assess 2 reports, one evaluating surgical procedures and the other evaluating participative interventions.

**Primary outcome**: The primary outcome will be the rate of correct answers for each group compared to a gold standard.

This gold standard will be determined by a consensus of 3 reviewers (LF, IB, PR) who will independently assess the panel of reports that will be evaluated by the participants.

This panel of reports will be randomized among all randomized control trials assessing nonpharmacological treatments and published in 2005-2006 in the following journals:

New England Journal of Medicine, JAMA, Lancet, Annals of Internal Medicine, BMJ,

Annals of Surgery, British Journal of Surgery, Annals of Surgical Oncology, Archives of General Psychiatry, American Journal of Psychiatry, Journal of Clinical Psychiatry, Physical Therapy, Supportive Care in Cancer, Archives of Physical Medicine and Rehabilitation

We will randomise reports, ensuring that half will concern surgical treatments and half participative interventions such as psychotherapy, rehabilitation, or diet

**Secondary outcome**: -rate of correct answers for each item

-qualitative assessment of knowledge-based expert system

**Sample size calculation**:

According to the literature, we expect a rate of correct answers of 0.75 (SD: 0.15) in the control arm.^26^

10 participants are necessary in each arm to demonstrate a difference of 0.15 with a power of 80% at the 0.05 level of significance.

The sample size was raised to 20 participants per arm considering the clustering effect for readers and the rate of possible non-response after randomisation.

The statistical analysis will involve a random effects model to take into account the reader effect and the article effect.

**D) Conclusion**

If effective, this program could be offered freely on the internet; and this work could lead to consider this kind of teaching strategy for other scales or check-lists to improve their reliability.

**Bibliography**
