# Supplementary material for: Development and Evaluation of a Pedagogical Tool to Improve Understanding of a Quality Checklist: A Randomised Controlled Trial
Source: PLoS Clin Trials. 2007 May 4;2(5):e22. doi: 10.1371/journal.pctr.0020022 (PMC1865084; doi:10.1371/journal.pctr.0020022)
Supplement: Trial Protocol — (28 KB DOC) [file pctr.0020022.sd003.doc]

Does a computer learning system improve the understanding and dissemination of CLEAR NPT?

## Protocol 2

# Lola Fourcade, Isabelle Boutron, David Moher, Peter Tugwell, Philippe Ravaud

Some modifications to the protocol occurred after the presentation of this protocol to the Scientific Committee of the Master of Methodology for treatment assessment of the University of Paris 7.

**Study design**: Paired randomised controlled trial comparing two groups of participants:

-intervention group: trained with the computer learning system,

-control group: no specific training

**Participants**:

The Cochrane Collaboration refused to participate in this survey. Consequently, we invited corresponding authors of meta-analyses of NPTs published between January 1, 2004, and March 3, 2006, the members of the HTAi group and the Directors of Evidence-based Practice Centers (EPC) to participate.

**Primary outcome**: Participants will be randomized by pairs so that they would be evaluated in the end on the same report. The primary outcome will be the rate of correct answers for each group compared to a gold standard.

**Sample size calculation**:

38 pairs of participants in each arm are necessary to demonstrate a difference of 0.10 with a power of 85% at the 0.05 level of significance using a paired Student’s *t*-test.

A sample size of 38 pairs will have 85% statistical power to detect a difference of 10% in means (e.g., a mean rate of correct responses of 70% in the intervention group and 60% in the control group), assuming a standard deviation of differences of 20%, using a paired Student’s *t*-test with a 0.05 two-sided significance level.

The mean rate of correct answers of participants will be compared to the criterion standard by paired Student’s *t*-test. The “per item rate” of correct answers to the criterion standard will be compared by use of a Mc Nemar test for paired dichotomous data and with Yates correction as appropriate. A p ≤ 0.05 will be considered significant, and all tests will be two-sided.
