# Supplementary material for: Development and Evaluation of a Pedagogical Tool to Improve Understanding of a Quality Checklist: A Randomised Controlled Trial
Source: PLoS Clin Trials. 2007 May 4;2(5):e22. doi: 10.1371/journal.pctr.0020022 (PMC1865084; doi:10.1371/journal.pctr.0020022)
Supplement: Figure S1 — (43 KB DOC) [file pctr.0020022.sg001.doc]

**Figure S1. Main principles of the Internet-based computer learning system**

Relevant passage of a report is proposed to participants.

Then, they must answer one item.

Wrong answer Right answer Explanation of the answer

based on the user guide

previously given to participants

A sub-item is to enhance memorization of the item

proposed to participants and its possible answers.

to improve the understanding

of the previous item

This sub-item can be a list of Second item is proposed

questions giving details about

the first item (i.e., what question

should be asked when reading

the first item).

Right answer to Wrong answer to the sub-item

the sub-item

User guide slide Another Explanation of the answers of items

explanation of the sub-item is AND sub-items based on the user

correct answer for designed guide

this example, then

go to the next item

User guide slide explanation

for this item

then go to the next item
