# Supplementary material for: Development and Evaluation of a Pedagogical Tool to Improve Understanding of a Quality Checklist: A Randomised Controlled Trial
Source: PLoS Clin Trials. 2007 May 4;2(5):e22. doi: 10.1371/journal.pctr.0020022 (PMC1865084; doi:10.1371/journal.pctr.0020022)
Supplement: Text S1 — (43 KB DOC) [file pctr.0020022.sd004.doc]

Text S1: A checklist to evaluate a report of a nonpharmacological trial (CLEAR NPT)

1. **Was the generation of allocation sequences adequate?** Yes / No / Unclear
2. **Was the treatment allocation concealed?** Yes / No / Unclear
3. **Were full details of the intervention administered to each group made available?** Yes / No / Unclear
4. **Were care providers’ experience or skill in each arm appropriate?** Yes / No / Unclear
5. **Was participant (i.e., patients) adherence assessed quantitatively?** Yes / No / Unclear
6. **Were participants adequately blinded?** Yes /

No, because blinding is not feasible /

No, although blinding is feasible /

Unclear

- 1. **If participants were not adequately blinded**
     1. **Were all other treatments and care (i.e., co-interventions) the same in each randomized group?** Yes / No / Unclear
     2. **Were withdrawals and dropout the same in each randomized group?** Yes / No / Unclear

1. **Were care providers or persons caring for the participants adequately blinded?** Yes /

No, because blinding is not feasible /

No, although blinding is feasible /

Unclear

- 1. **If care providers were not adequately blinded**
     1. **Were all other treatments and care (i.e., co-interventions) the same in each randomized group?** Yes / No / Unclear
     2. **Were withdrawals and dropout the same in each randomized group?** Yes / No / Unclear

1. **Were outcome assessors adequately blinded to assess the primary outcomes?** Yes /

No, because blinding is not feasible /

No, although blinding is feasible /

Unclear

- 1. **If outcome assessors were not adequately blinded, were specific methods used to avoid ascertainment bias (systematic differences in outcome assessment)**  Yes / No / Unclear

1. **Was the follow-up schedule the same in each group?** Yes / No / Unclear
2. **Were the main outcomes analyzed according to the intention-to-treat principle?** Yes / No / Unclear
