# Supplementary material for: Development and Evaluation of a Pedagogical Tool to Improve Understanding of a Quality Checklist: A Randomised Controlled Trial
Source: PLoS Clin Trials. 2007 May 4;2(5):e22. doi: 10.1371/journal.pctr.0020022 (PMC1865084; doi:10.1371/journal.pctr.0020022)
Supplement: Text S2 — (82 KB DOC) [file pctr.0020022.sd005.doc]

**Text S2: User guide for the checklist of items assessing the quality of randomized controlled trials of nonpharmacological treatment**

Reviewers answer the following items, taking into account the data reported in the article or obtained from the study’s authors.

**1. Was the generation of allocation sequences adequate?**

a) Modality of answering: Yes / No / Unclear

;;

b) Context: Empirical studies showed that inadequate generation of allocation sequences could yield inflated treatment effect. Adequate generation of allocation sequences is necessary to avoid bias.

c) How to assess this item:

- - The answer should be “**yes**” for this item if the method used to generate the sequence of randomization is suitable to prevent selection bias by use of, for example, a table of random numbers, random numbers generated by computer, coin tossing or shuffling cards.
  - The answer should be “**no**” for this item if the method is described but inadequate, i.e., if sequences could be related to prognosis and thus introduce selection bias.
  - The answer should be “**unclear**” for this item if the method is not described or there is insufficient information to make a judgment.

**2.Was the treatment allocation concealed?**

a) Modality of answering: Yes / No / Unclear

b) Context: Empirical studies showed that inadequate or unclear allocation concealment could yield inflated treatment effect. Adequate allocation concealment is necessary to avoid bias.

c) How to assess this item:

- - The answer should be **“yes”** for this item if patients and investigators enrolling patients could not foresee treatment assignments by use of, for example, centralized randomization, opaque sealed envelopes, numbered or coded devices.
  - The answer should be **“no”** for this item if the method is described but inadequate, i.e., if patients and investigators enrolling patients could foresee assignments. For example, procedures based on inadequate generation of allocation sequences, open allocation schedule, unsealed or non-opaque envelopes.
  - The answer should be **“unclear”** for this item if there is insufficient information to make a judgment.

**3. Were details of the intervention administered to each group made available?**

a) Modality of answering: Yes / No / Unclear

b) What is meant by this item:

To be able to assess what the treatment administered was and to be able to consider two interventions similar enough to be grouped in a meta-analysis, clear descriptions of the following data should be reported or made available:

- procedure provided by care providers (e.g., details on frequency of administration, time to complete treatment, total duration of treatment, indication for initiation of treatment, modification of the treatment procedure, discontinuation of the treatment and source of equipment),
- general principles of the individualization procedure when necessary (i.e., clear description of treatment modifications according to defined criteria such as individuals’ tolerance, co-morbidities and/or treatment efficacy. For example, for an exercise therapy, exercises were tailored to the participant’s ability to perform them without pain (exercise intensity was reduced or type of exercise was modified).

c) How to assess this item:

- - The answer should be “yes” for this item if these data were either described in the report or made available (reference to a preliminary report, online addendum etc.). For example, the answer should be “yes” if details on treatment were described as follows: “Rheumatologists in primary care settings were instructed to perform the treatment they usually provide to participants with osteoarthritis”, and if in the results section, details on the treatment provided are described in a table with the pharmacological treatments and nonpharmacological treatments provided.
  - The answer should be “**no**” for this item if these descriptions were neither **described in the report** nor **made available** (reference to a preliminary report, online addendum etc.).
  - The answer should be “**unclear**” for this item if these descriptions were partially reported and insufficient to assess this item as “yes.”

**4. Were care providers’ experience or skill in each arm appropriate?**

a) Modality of answering: Yes / No / Unclear

b) What is meant by this item:

Care providers’ experience or skill will be assessed only **for** **therapist-dependent interventions** (i.e., interventions where the success of the treatment are directly linked to care providers’ technical skill and where care providers are part of the treatment procedure). For other treatment, this item is not relevant and should be removed from the checklist or answered “unclear”.

It is important to assess care providers’ experience or skill to avoid bias. For example, when comparing surgery A to surgery B, if surgeons have performed several surgery As but do not have the experience or skill necessary to adequately perform surgery B, the trial results will be biased in favor of A.

Appropriate experience or skill is not necessarily similar in each study arm, particularly when the interventions compared are different (e.g., surgery versus rehabilitation) and making judgments about appropriate experience or skill may be difficult. Appropriate experience or skill should be determined according to published data, preliminary studies, guidelines, run-in period or, if these data are lacking, a group of experts. Appropriate experience or skill will have to be determined and prespecified in the protocol for each study arm before the beginning of the survey.

In efficacy trials, care providers’ **skill** should be appropriate. For example, in an efficacy trial assessing carotid endarterectomy, each surgeon had their last 50 operations assessed and if more than 2 resulted in complications they could not join the trial.

In pragmatic trials, care providers’ **experience** should be appropriate (number of interventions performed or years of practice or training).

How to assess this item:

- - The answer should be “**yes**” for this item if care providers’ experience or skill was appropriate in each arm according to the prespecified standard.
  - The answer should be “**no**” for this item if care providers’ experience or skill was inappropriate according to the prespecified standard in at least one arm.
  - The answer should be “**unclear**” for this item if there is insufficient information to make a judgment.

**5.** **Was participant (i.e., patients) adherence assessed quantitatively?**

a) Modality of answering: Yes / No / Unclear

b) What is meant by this item:

Treatment adherence will be assessed only **for treatments necessitating iterative interventions** (e.g., physiotherapy that supposes several sessions, in contrast to a “one-shot” treatment such as surgery, in which treatment adherence is not assessed quantitatively). For one-shot treatment, this item is not relevant and should be removed from the checklist or answered “unclear”.

Participant adherence assessed quantitatively could be, for example, the number of physiotherapy sessions attended by participants.

In efficacy trials, participant adherence should be closely monitored with compliance-improving strategies for participants.

In pragmatic trials, participant adherence should be monitored unobtrusively with no enhancing interventions)

c) How to assess this item:

- - The answer should be “**yes**” for this item if participants’ adherence to the treatment is reported.
  - The answer should be “**no**” for this item if participants’ adherence to the treatment is not reported.
  - The answer should be “**unclear**” for this item if there is insufficient information to make a judgment.

**6.** **Were participants adequately blinded?**

a) Modality of answering: Yes / No, because blinding is not feasible / No, although blinding is feasible / Unclear

b) What is meant by this item:

Lack of blinding has shown to be potentially associated with bias. However, in NPT trials, blinding is often impossible, and the feasibility of blinding should be taken into account.

c) How to assess this item:

- - The answer should be “**yes**” for this item, if
    - the control treatment’s characteristics are indistinguishable from the experimental treatment for participants (i.e., the treatments, consequences of the treatments [adverse effects, clinical manifestations during treatment administration] and monitoring are similar in each group)

**or**

- - - the success of blinding was tested and successful in a preliminary study before the trial was performed. In fact, breaking the blind can be linked to a beneficial effect of treatments, and assessment of blinding success would be more reliable in trials if carried out before clinical outcome has been determined (i.e., in preliminary studies).
  - The answer should be “**no**, **because blinding is not feasible**” for this item if participants were not blinded because the treatments being compared are totally different and the use of double dummy is impossible (e.g., comparison of surgery to physiotherapy) and a creative solution to blind participants has never been found.
  - The answer should be “**no**, **although blinding is feasible**” for this item if participants were not blinded, although blinding participants has been previously performed successfully.
  - The answer should be “**unclear**” for this item if there is insufficient information to make a judgment.

**6.1.** **If participants were not adequately blinded,**

**6.1.1 Were all other treatments and care (i.e., co-interventions) the same in each randomized group?**

**6.1.2 Were withdrawals and lost to follow-up the same in each randomized group?**

a) Modality of answering: Yes / No / Unclear

b) What is meant by these items:

Co-interventions = provision of unintended additional care to either comparison group. All co-interventions that may plausibly impact the outcome should be administered more or less equally in treatment and control groups.

Lost to follow-up, withdrawal and use of co-intervention can be linked to treatment failure and could be used as outcomes. Thus, it could be difficult to conclude that a trial had low quality on these items when, in fact, these outcomes could be linked to a beneficial effect in one group. Nevertheless, when participants are not blinded, performance bias (i.e., unequal provision of care apart from the treatment under evaluation) and attrition bias could occur, because knowing treatment allocation could modify the use of co-interventions and participants’ willingness to adhere to the survey and, therefore, bias the results. One would probably be more confident with the results of a trial in which participants were not blinded but co-interventions, withdrawals and lost to follow-ups were similar in all arms.

c) How to assess these items

Item 6.1.1:

If participants were blinded, this item is not relevant and should be removed from the checklist.

- - The answer should be “**yes**” for this item if co-interventions were similar in both groups.
  - The answer should be “**no**” for this item if co-interventions were not similar in both groups and could modify the treatment effect.
  - The answer should be “**unclear**” for this item if there is insufficient information to make a judgment.

Item 6.1.2:

If participants were blinded, this item is not relevant and should be removed from the checklist

- - The answer should be “**yes**” for this item if the number and description of withdrawals and lost to follow-up are similar in both groups.
  - The answer should be “**no**” for this item if the number and / or description of withdrawals and lost to follow-ups are not similar in both groups and could modify the treatment effect.
  - The answer should be “**unclear**” for this item if there is insufficient information to make a judgment.

**7.** **Were care providers or people caring for the participants adequately blinded?**

a) Modality of answering: Yes / No, because blinding is not feasible / No, although blinding is feasible / Unclear

b) What is meant by this item:

Lack of blinding has shown to be potentially associated with bias. However, in NPT trials, blinding is often impossible, and the feasibility of blinding should be taken into account.

**Care providers** = People administering the intervention: physicians, nurses, physiotherapists, surgeons etc. For example, in ultrasound therapy, care providers can be blinded by a sham device that is switched off.

**Persons caring for participants** = people administering co-interventions and following participants. In some situations (when later care does not require handling complications of earlier care and when participant care is not compromised), care providers are not involved in follow-up decisions about further treatments. For example, when assessing joint lavage versus placebo, the person caring for patients was a blinded rheumatologist who did not perform the lavage (Bradley, Arthritis Rheum, 2002).

c) How to assess this item:

- - The answer should be “**yes**” for this item if
- the control treatment’s characteristics are indistinguishable from the experimental treatment for care providers or the people caring for patients (i.e., the treatments, consequences of the treatments [adverse effects, clinical manifestations during treatment administration] and monitoring are similar in each group).

**or**

- - the success of blinding was tested and successful in a preliminary study before the trial was performed. In fact, breaking the blind can be linked to a beneficial effect of treatments, and assessment of blinding success would be more reliable in trials if carried out before clinical outcome has been determined (i.e., in preliminary studies).
  - The answer should be “**no**, **because blinding is not feasible**” for this item if care providers and the people caring for patients were not blinded because the treatments being compared are totally different and the use of double dummy is impossible (e.g., comparison of surgery to physiotherapy) and a creative solution to blind care providers or the people caring for patients has never been found.
  - The answer should be “**no**, **although blinding is feasible**” for this item if care providers or the people caring for patients were not blinded, although blinding care providers or the people caring for patients was previously performed successfully.
  - The answer should be “**unclear**” for this item if there is insufficient information to make a judgment.

**7.1.** **If care providers were not adequately blinded,**

**7.1.1 Were all other treatments and care (i.e., co-interventions) the same in each randomized group?**

**7.1.2 Were withdrawals and lost to follow-up the same in each randomized group?**

a) Modality of answering: Yes / No / Unclear

b) What is meant by these items:

Co-interventions = provision of unintended additional care to either comparison group. All co-interventions that may plausibly affect the outcome should be administered more or less equally in treatment and control groups.

Lost to follow-up, withdrawals and co-interventions can be linked to treatment failure and could be used as outcomes. Thus, it could be difficult to conclude that a trial had low quality on these items when, in fact, these outcomes could be linked to a beneficial effect in one group. Nevertheless, when care providers are not blinded, performance bias (i.e., unequal provision of care apart from the treatment under evaluation) and attrition bias could occur, because knowing treatment allocation could modify the use of co-interventions and care providers’ willingness to maintain their patients in the survey and, therefore, bias the results. One would probably be more confident with the results of a trial in which care providers were not blinded, but co-interventions, withdrawals and lost to follow-up were similar in all arms.

c) How to assess these items

Item 7.1.1:

If care providers were blinded, these items are not relevant and should be removed from the checklist

- - The answer should be “**yes**” for this item if co-interventions were similar in both groups.
  - The answer should be “**no**” for this item if co-interventions were not similar in both groups and could modify the treatment effect.
  - The answer should be “**unclear**” for this item if there is insufficient information to make a judgment.

Item 7.1.2

If care providers were blinded, this item is not relevant and should be removed from the checklist

- - The answer should be “**yes**” for this item if the number and description of withdrawals and lost to follow-ups are similar in both groups.
  - The answer should be “**no**” for this item if the number and description of withdrawals and lost to follow-up are not similar in both groups and could modify the treatment effect.
  - The answer should be “**unclear**” for this item if there is insufficient information to make a judgment.

**8. Were outcome assessors adequately blinded to assess the primary outcomes?**

a) Modality of answering: Yes / No, because blinding is not feasible / No, although blinding is feasible / Unclear

b) What is meant by this item:

**Outcome assessors** = people assessing participants’ outcomes. Outcome assessors could be participants [self-reported outcomes such as pain], data collectors [blood pressure], blinded radiologists [radiographic assessment]

Adequacy of blinding should be assessed for the **primary outcomes**.

c) How to assess this item:

- The answer should be “**yes**” for this item, if
- the success of blinding was tested in **a preliminary study** before the trial is performed. In fact, breaking the blind can be linked to a beneficial effect of treatments, and assessment of blinding success would be more reliable in trials if carried out before the clinical outcome has been determined (i.e., in preliminary studies).

**or**

- the adequacy of the blinding procedure will depend on the main outcome:
  - **for participant-reported outcomes** in which the participant is the outcome assessor (e.g., pain, disability): the blinding procedure is adequate for outcome assessors if the blinding procedure is adequate for participants (i.e., the control treatment’s characteristics are indistinguishable from the experimental treatment for participants)
  - **for outcome criteria assessed during scheduled visit and that suppose a contact between participants and outcome assessors** (e.g., clinical examination): the blinding procedure is adequate if participants are blinded, and the treatment or adverse effects of the treatment cannot be noticed during clinical examination
  - **for outcome criteria that do not suppose a contact with participants** (e.g., radiography, magnetic resonance imaging, electrocardiography): the blinding procedure is adequate if the treatment or adverse effects of the treatment cannot be noticed when assessing the main outcome
  - **for outcome criteria that are clinical or therapeutic events** that will be determined by the interaction between participants and care providers (e.g., co-interventions, hospitalization length, recurrent stroke, treatment failure, intervention for total hip arthroplasty), in which the care provider is the outcome assessor: the blinding procedure is adequate for outcome assessors if the blinding procedure is adequate for care providers, (i.e., the control treatment’s characteristics are indistinguishable from the experimental treatment for care providers)
  - **for outcome criteria that are assessed from data of the medical forms**: the blinding procedure is adequate if the treatment or adverse effects of the treatment cannot be noticed on the extracted data
  - The answer should be “**no**, **because blinding is not feasible**” for this item if outcome assessors were not blinded because treatment or adverse effects of the treatment are obvious when assessing the main outcome (e.g., arthroplasty with cement versus no cement on a radiograph), and no creative solution was performed successfully to blind outcome assessors in such circumstances.
  - The answer should be “**no**, **although blinding is feasible** ” for this item if outcome assessors were not blinded, although blinding outcome assessors has been previously performed successfully.
  - The answer should be “**unclear**” for this item if there is insufficient information to make a judgment.

**8.1.** **If outcome assessors were not adequately blinded, were specific methods used to avoid ascertainment bias (systematic differences in outcome assessment).**

a) Modality of answering: Yes / No / Unclear

b) What is meant by this item:

Ascertainment bias (systematic differences in outcome assessment) can be avoided or restricted if the main outcome is hard or objective (e.g., death), a blinded or at least independent committee was responsible for the adjudication of primary outcomes, outcome assessment was carefully standardized and performed by an independent assessor or outcome assessors were blinded to the study hypothesis.

c) How to assess this item:

If outcome assessors were blinded, this item is not relevant and should be removed from the checklist

- The answer should be “**yes**” for this item, if
- **the main outcome is objective** or **“hard”** (blinding is particularly important for subjective or “soft” outcomes such as pain but less important for objective or “hard” criteria such as death)

**or**

- **outcomes were assessed by a blinded or at least an independent endpoint review committee** (independent committee assessing whether a participant meets the criteria for a study’s outcome. For example, this committee could judge treatment failure, death linked to myocardial infarction, etc.)

**or**

- **outcomes were assessed by an independent outcome assessor trained to perform the measurements in a standardized manner**

**or**

- **the outcome assessor was blinded to the study purpose and hypothesis** (e.g., to investigate whether breast feeding is effective for pain relief during venepuncture, 2 specially trained observers independently assessed a videotape of the child. Observers were blinded to the purpose and hypothesis of the study, because they had been told that the study assessed the reproducibility of their assessment in different situations (Carbajal, BMJ. 2003)
- The answer should be “**no**” for this item if no effort was made to avoid ascertainment bias.
- The answer should be “**unclear**” for this item if there is insufficient information to make a judgment.

**9.** **Was the follow-up schedule the same in each group?**

a) Modality of answering: Yes / No / Unclear

b)What is meant by this item:

This item is not relevant for trials in which follow-up is part of the question. For example, this item is not relevant for a trial assessing frequent versus less frequent follow-up for cancer recurrence. In these situations, this item should be removed from the checklist or answered “unclear”.

c) How to assess this item:

- - The answer should be “**yes**” for this item if the actual prospectively defined schedule for follow-up visits is similar in each group.
  - The answer should be “**no**” for this item if the actual prospectively defined schedule for follow-up visits is not similar in each group.
  - The answer should be “**unclear**” for this item if there is insufficient information to make a judgment.

**10. Were the main outcomes analyzed according to the intention-to-treat principle?**

a) Modality of answering: Yes / No / Unclear

b) What is meant by this item:

The intention-to-treat principle means that all participants randomized were included in the analysis and kept in their original group.

c) How to assess this item:

- - The answer should be “**yes**” for this item if all participants randomized were included in the analysis and kept in their original group.
  - The answer should be “**no**” for this item if all participants randomized were not included in the analysis or were not kept in their original group.
  - The answer should be “**unclear**” for this item if there is insufficient information to make a judgment.
