# Supplementary material for: Development and Evaluation of a Pedagogical Tool to Improve Understanding of a Quality Checklist: A Randomised Controlled Trial
Source: PLoS Clin Trials. 2007 May 4;2(5):e22. doi: 10.1371/journal.pctr.0020022 (PMC1865084; doi:10.1371/journal.pctr.0020022)
Supplement: Text S3 — (50 KB DOC) [file pctr.0020022.sd006.doc]

Text S3: Panel of reports used to develop the computer learning system.

1. Appel LJ, Champagne CM, Harsha DW, et al. Effects of comprehensive lifestyle modification on blood pressure control: main results of the PREMIER clinical trial. *Jama.* Apr 23-30 2003;289(16):2083-2093.

2. Bikhchandani J, Agarwal PN, Kant R, Malik VK. Randomized controlled trial to compare the early and mid-term results of stapled versus open hemorrhoidectomy. *Am J Surg.* Jan 2005;189(1):56-60.

3. Brealey S, Burton K, Coulton S, et al. UK Back pain Exercise And Manipulation (UK BEAM) trial--national randomised trial of physical treatments for back pain in primary care: objectives, design and interventions [ISRCTN32683578]. *BMC Health Serv Res.* Aug 1 2003;3(1):16.

4. Brown GK, Ten Have T, Henriques GR, Xie SX, Hollander JE, Beck AT. Cognitive therapy for the prevention of suicide attempts: a randomized controlled trial. *Jama.* Aug 3 2005;294(5):563-570.

5. Chang RW, Falconer J, Stulberg SD, Arnold WJ, Manheim LM, Dyer AR. A randomized, controlled trial of arthroscopic surgery versus closed-needle joint lavage for patients with osteoarthritis of the knee. *Arthritis Rheum.* Mar 1993;36(3):289-296.

6. Chintamani, Singhal V, Singh J, Bansal A, Saxena S. Half versus full vacuum suction drainage after modified radical mastectomy for breast cancer - a prospective randomized clinical trial[ISRCTN24484328]. *BMC Cancer.* Jan 27 2005;5(1):11.

7. Collins CT, Ryan P, Crowther CA, McPhee AJ, Paterson S, Hiller JE. Effect of bottles, cups, and dummies on breast feeding in preterm infants: a randomised controlled trial. *Bmj.* Jul 24 2004;329(7459):193-198.

8. Cushnaghan J, McCarthy C, Dieppe P. Taping the patella medially: a new treatment for osteoarthritis of the knee joint? *Bmj.* Mar 19 1994;308(6931):753-755.

9. Dansinger ML, Gleason JA, Griffith JL, Selker HP, Schaefer EJ. Comparison of the Atkins, Ornish, Weight Watchers, and Zone diets for weight loss and heart disease risk reduction: a randomized trial. *Jama.* Jan 5 2005;293(1):43-53.

10. Deyle GD, Henderson NE, Matekel RL, Ryder MG, Garber MB, Allison SC. Effectiveness of manual physical therapy and exercise in osteoarthritis of the knee. A randomized, controlled trial. *Ann Intern Med.* Feb 1 2000;132(3):173-181.

11. Dobkin B, Apple D, Barbeau H, et al. Weight-supported treadmill vs over-ground training for walking after acute incomplete SCI. *Neurology.* Feb 28 2006;66(4):484-493.

12. Doukas G, Samani NJ, Alexiou C, et al. Left atrial radiofrequency ablation during mitral valve surgery for continuous atrial fibrillation: a randomized controlled trial. *Jama.* Nov 9 2005;294(18):2323-2329.

13. Elden H, Ladfors L, Olsen MF, Ostgaard HC, Hagberg H. Effects of acupuncture and stabilising exercises as adjunct to standard treatment in pregnant women with pelvic girdle pain: randomised single blind controlled trial. *Bmj.* Apr 2 2005;330(7494):761.

14. Ettinger WH, Jr., Burns R, Messier SP, et al. A randomized trial comparing aerobic exercise and resistance exercise with a health education program in older adults with knee osteoarthritis. The Fitness Arthritis and Seniors Trial (FAST). *Jama.* Jan 1 1997;277(1):25-31.

15. Frost H, Lamb SE, Doll HA, Carver PT, Stewart-Brown S. Randomised controlled trial of physiotherapy compared with advice for low back pain. *Bmj.* Sep 25 2004;329(7468):708.

16. Halbert J, Crotty M, Weller D, Ahern M, Silagy C. Primary care-based physical activity programs: effectiveness in sedentary older patients with osteoarthritis symptoms. *Arthritis Rheum.* Jun 2001;45(3):228-234.

17 Hannah ME, Hannah WJ, Hodnett ED, et al. Outcomes at 3 months after planned cesarean vs planned vaginal delivery for breech presentation at term: the international randomized Term Breech Trial. *Jama.* Apr 10 2002;287(14):1822-1831.

18. Heshka S, Anderson JW, Atkinson RL, et al. Weight loss with self-help compared with a structured commercial program: a randomized trial. *Jama.* Apr 9 2003;289(14):1792-1798.

19. Hubbard MJ. Articular debridement versus washout for degeneration of the medial femoral condyle. A five-year study. *J Bone Joint Surg Br.* Mar 1996;78(2):217-219.

20. Hunkeler EM, Katon W, Tang L, et al. Long term outcomes from the IMPACT randomised trial for depressed elderly patients in primary care. *Bmj.* Feb 4 2006;332(7536):259-263.

21. Kirkley A, Webster-Bogaert S, Litchfield R, et al. The effect of bracing on varus gonarthrosis. *J Bone Joint Surg Am.* Apr 1999;81(4):539-548.

22. Kosaka K, Noda M, Kuzuya T. Prevention of type 2 diabetes by lifestyle intervention: a Japanese trial in IGT males. *Diabetes Res Clin Pract.* Feb 2005;67(2):152-162.

23. Kumar S, Jain S. Treatment of appendiceal mass: prospective, randomized clinical trial. *Indian J Gastroenterol.* Sep-Oct 2004;23(5):165-167.

24. Linde K, Streng A, Jurgens S, et al. Acupuncture for patients with migraine: a randomized controlled trial. *Jama.* May 4 2005;293(17):2118-2125.

25. Little P, Kelly J, Barnett J, Dorward M, Margetts B, Warm D. Randomised controlled factorial trial of dietary advice for patients with a single high blood pressure reading in primary care. *Bmj.* May 1 2004;328(7447):1054.

26. Martin BI, Levenson LM, Hollingworth W, et al. Randomized clinical trial of surgery versus conservative therapy for carpal tunnel syndrome [ISRCTN84286481]. *BMC Musculoskelet Disord.* 2005;6(1):2.

27. McInnes J, Larson MG, Daltroy LH, et al. A controlled evaluation of continuous passive motion in patients undergoing total knee arthroplasty. *Jama.* Sep 16 1992;268(11):1423-1428.

28. Nakamura E, Mizuta H, Kudo S, Takagi K, Sakamoto K. Open-wedge osteotomy of the proximal tibia hemicallotasis. *J Bone Joint Surg Br.* Nov 2001;83(8):1111-1115.

29. Onsten I, Carlsson AS, Ohlin A, Nilsson JA. Migration of acetabular components, inserted with and without cement, in one-stage bilateral hip arthroplasty. A controlled, randomized study using roentgenstereophotogrammetric analysis. *J Bone Joint Surg Am.* Feb 1994;76(2):185-194.

30. Patchell RA, Tibbs PA, Regine WF, et al. Postoperative radiotherapy in the treatment of single metastases to the brain: a randomized trial. *Jama.* Nov 4 1998;280(17):1485-1489.

31. Puskas JD, Williams WH, Mahoney EM, et al. Off-pump vs conventional coronary artery bypass grafting: early and 1-year graft patency, cost, and quality-of-life outcomes: a randomized trial. *Jama.* Apr 21 2004;291(15):1841-1849.

32. Quinn J, Cummings S, Callaham M, Sellers K. Suturing versus conservative management of lacerations of the hand: randomised controlled trial. *Bmj.* Aug 10 2002;325(7359):299.

33. Singh RB, Dubnov G, Niaz MA, et al. Effect of an Indo-Mediterranean diet on progression of coronary artery disease in high risk patients (Indo-Mediterranean Diet Heart Study): a randomised single-blind trial. *Lancet.* Nov 9 2002;360(9344):1455-1461.

34. Toda Y, Segal N, Kato A, Yamamoto S, Irie M. Effect of a novel insole on the subtalar joint of patients with medial compartment osteoarthritis of the knee. *J Rheumatol.* Dec 2001;28(12):2705-2710.

35. Van Baar ME, Dekker J, Oostendorp RA, et al. The effectiveness of exercise therapy in patients with osteoarthritis of the hip or knee: a randomized clinical trial. *J Rheumatol.* Dec 1998;25(12):2432-2439.

36. van Staaij BK, van den Akker EH, Rovers MM, Hordijk GJ, Hoes AW, Schilder AG. Effectiveness of adenotonsillectomy in children with mild symptoms of throat infections or adenotonsillar hypertrophy: open, randomised controlled trial. *Bmj.* Sep 18 2004;329(7467):651.

37. Vas J, Mendez C, Perea-Milla E, et al. Acupuncture as a complementary therapy to the pharmacological treatment of osteoarthritis of the knee: randomised controlled trial. *Bmj.* Nov 20 2004;329(7476):1216.

38. Vickers AJ, Rees RW, Zollman CE, et al. Acupuncture for chronic headache in primary care: large, pragmatic, randomised trial. *Bmj.* Mar 27 2004;328(7442):744.

39. Viljanen M, Malmivaara A, Uitti J, Rinne M, Palmroos P, Laippala P. Effectiveness of dynamic muscle training, relaxation training, or ordinary activity for chronic neck pain: randomised controlled trial. *Bmj.* Aug 30 2003;327(7413):475.

40. Wiebe S, Blume WT, Girvin JP, Eliasziw M. A randomized, controlled trial of surgery for temporal-lobe epilepsy. *N Engl J Med.* Aug 2 2001;345(5):311-318.
