# Supplementary material for: Development and Evaluation of a Pedagogical Tool to Improve Understanding of a Quality Checklist: A Randomised Controlled Trial
Source: PLoS Clin Trials. 2007 May 4;2(5):e22. doi: 10.1371/journal.pctr.0020022 (PMC1865084; doi:10.1371/journal.pctr.0020022)
Supplement: Text S4 — (51 KB DOC) [file pctr.0020022.sd007.doc]

**Text S4: Panel of reports used for the final assessment of participants**

**1.** Effectiveness of treatment for alcohol problems: findings of the randomised UK alcohol treatment trial (UKATT). *Bmj.* Sep 10 2005;331(7516):541.

**2.** Acosta JM, Katkhouda N, Debian KA, Groshen SG, Tsao-Wei DD, Berne TV. Early ductal decompression versus conservative management for gallstone pancreatitis with ampullary obstruction: a prospective randomized clinical trial. *Ann Surg.* Jan 2006;243(1):33-40.

**3.** Ada L, Goddard E, McCully J, Stavrinos T, Bampton J. Thirty minutes of positioning reduces the development of shoulder external rotation contracture after stroke: a randomized controlled trial. *Arch Phys Med Rehabil.* Feb 2005;86(2):230-234.

**4.** Adam DJ, Beard JD, Cleveland T, et al. Bypass versus angioplasty in severe ischaemia of the leg (BASIL): multicentre, randomised controlled trial. *Lancet.* Dec 3 2005;366(9501):1925-1934.

**5.** Anthonisen NR, Skeans MA, Wise RA, Manfreda J, Kanner RE, Connett JE. The effects of a smoking cessation intervention on 14.5-year mortality: a randomized clinical trial. *Ann Intern Med.* Feb 15 2005;142(4):233-239.

**6.** Appel LJ, Sacks FM, Carey VJ, et al. Effects of protein, monounsaturated fat, and carbohydrate intake on blood pressure and serum lipids: results of the OmniHeart randomized trial. *Jama.* Nov 16 2005;294(19):2455-2464.

**7.** Assefi NP, Sherman KJ, Jacobsen C, Goldberg J, Smith WR, Buchwald D. A randomized clinical trial of acupuncture compared with sham acupuncture in fibromyalgia. *Ann Intern Med.* Jul 5 2005;143(1):10-19.

**8.** Baigrie RJ, Cullis SN, Ndhluni AJ, Cariem A. Randomized double-blind trial of laparoscopic Nissen fundoplication versus anterior partial fundoplication. *Br J Surg.* Jul 2005;92(7):819-823.

**9.** Bill-Axelson A, Holmberg L, Ruutu M, et al. Radical prostatectomy versus watchful waiting in early prostate cancer. *N Engl J Med.* May 12 2005;352(19):1977-1984.

**10.** Blankensteijn JD, de Jong SE, Prinssen M, et al. Two-year outcomes after conventional or endovascular repair of abdominal aortic aneurysms. *N Engl J Med.* Jun 9 2005;352(23):2398-2405.

**11.** Blumenthal JA, Sherwood A, Babyak MA, et al. Effects of exercise and stress management training on markers of cardiovascular risk in patients with ischemic heart disease: a randomized controlled trial. *Jama.* Apr 6 2005;293(13):1626-1634.

**12.** Cauza E, Hanusch-Enserer U, Strasser B, et al. The relative benefits of endurance and strength training on the metabolic factors and muscle function of people with type 2 diabetes mellitus. *Arch Phys Med Rehabil.* Aug 2005;86(8):1527-1533.

**13.** Cengiz Y, Janes A, Grehn A, Israelsson LA. Randomized trial of traditional dissection with electrocautery versus ultrasonic fundus-first dissection in patients undergoing laparoscopic cholecystectomy. *Br J Surg.* Jul 2005;92(7):810-813.

**14.** Delis KT, Nicolaides AN. Effect of intermittent pneumatic compression of foot and calf on walking distance, hemodynamics, and quality of life in patients with arterial claudication: a prospective randomized controlled study with 1-year follow-up. *Ann Surg.* Mar 2005;241(3):431-441.

**15.** Ellis T, de Goede CJ, Feldman RG, Wolters EC, Kwakkel G, Wagenaar RC. Efficacy of a physical therapy program in patients with Parkinson's disease: a randomized controlled trial. *Arch Phys Med Rehabil.* Apr 2005;86(4):626-632.

**16.** Fairbank J, Frost H, Wilson-MacDonald J, Yu LM, Barker K, Collins R. Randomised controlled trial to compare surgical stabilisation of the lumbar spine with an intensive rehabilitation programme for patients with chronic low back pain: the MRC spine stabilisation trial. *Bmj.* May 28 2005;330(7502):1233.

**17.** Fassiadis N, Roidl M, Hennig M, South LM, Andrews SM. Randomized clinical trial of vertical or transverse laparotomy for abdominal aortic aneurysm repair. *Br J Surg.* Oct 2005;92(10):1208-1211.

**18.** Fitzgibbons RJ, Jr., Giobbie-Hurder A, Gibbs JO, et al. Watchful waiting vs repair of inguinal hernia in minimally symptomatic men: a randomized clinical trial. *Jama.* Jan 18 2006;295(3):285-292.

**19.** Gershlick AH, Stephens-Lloyd A, Hughes S, et al. Rescue angioplasty after failed thrombolytic therapy for acute myocardial infarction. *N Engl J Med.* Dec 29 2005;353(26):2758-2768.

**20.** Granholm E, McQuaid JR, McClure FS, et al. A randomized, controlled trial of cognitive behavioral social skills training for middle-aged and older outpatients with chronic schizophrenia. *Am J Psychiatry.* Mar 2005;162(3):520-529.

**21.** Guillou PJ, Quirke P, Thorpe H, et al. Short-term endpoints of conventional versus laparoscopic-assisted surgery in patients with colorectal cancer (MRC CLASICC trial): multicentre, randomised controlled trial. *Lancet.* May 14-20 2005;365(9472):1718-1726.

**22.** Hay EM, Mullis R, Lewis M, et al. Comparison of physical treatments versus a brief pain-management programme for back pain in primary care: a randomised clinical trial in physiotherapy practice. *Lancet.* Jun 11-17 2005;365(9476):2024-2030.

**23.** Johansson M, Thune A, Nelvin L, Stiernstam M, Westman B, Lundell L. Randomized clinical trial of open versus laparoscopic cholecystectomy in the treatment of acute cholecystitis. *Br J Surg.* Jan 2005;92(1):44-49.

**24.** Kennedy T, Jones R, Darnley S, Seed P, Wessely S, Chalder T. Cognitive behaviour therapy in addition to antispasmodic treatment for irritable bowel syndrome in primary care: randomised controlled trial. *Bmj.* Aug 20 2005;331(7514):435.

**25.** Kool JP, Oesch PR, Bachmann S, et al. Increasing days at work using function-centered rehabilitation in nonacute nonspecific low back pain: a randomized controlled trial. *Arch Phys Med Rehabil.* May 2005;86(5):857-864.

**26.** Lam DH, Hayward P, Watkins ER, Wright K, Sham P. Relapse prevention in patients with bipolar disorder: cognitive therapy outcome after 2 years. *Am J Psychiatry.* Feb 2005;162(2):324-329.

**27.** Lee WJ, Yu PJ, Wang W, Chen TC, Wei PL, Huang MT. Laparoscopic Roux-en-Y versus mini-gastric bypass for the treatment of morbid obesity: a prospective randomized controlled clinical trial. *Ann Surg.* Jul 2005;242(1):20-28.

**28.** Leroi AM, Parc Y, Lehur PA, et al. Efficacy of sacral nerve stimulation for fecal incontinence: results of a multicenter double-blind crossover study. *Ann Surg.* Nov 2005;242(5):662-669.

**29.** Maartense S, Dunker MS, Slors JF, et al. Laparoscopic-assisted versus open ileocolic resection for Crohn's disease: a randomized trial. *Ann Surg.* Feb 2006;243(2):143-149; discussion 150-143.

**30.** Martelli G, Boracchi P, De Palo M, et al. A randomized trial comparing axillary dissection to no axillary dissection in older patients with T1N0 breast cancer: results after 5 years of follow-up. *Ann Surg.* Jul 2005;242(1):1-6; discussion 7-9.

**31.** McIntosh VV, Jordan J, Carter FA, et al. Three psychotherapies for anorexia nervosa: a randomized, controlled trial. *Am J Psychiatry.* Apr 2005;162(4):741-747.

**32.** Melchart D, Streng A, Hoppe A, et al. Acupuncture in patients with tension-type headache: randomised controlled trial. *Bmj.* Aug 13 2005;331(7513):376-382.

**33.** Patchell RA, Tibbs PA, Regine WF, et al. Direct decompressive surgical resection in the treatment of spinal cord compression caused by metastatic cancer: a randomised trial. *Lancet.* Aug 20-26 2005;366(9486):643-648.

**34.** Prentice RL, Caan B, Chlebowski RT, et al. Low-fat dietary pattern and risk of invasive breast cancer: the Women's Health Initiative Randomized Controlled Dietary Modification Trial. *Jama.* Feb 8 2006;295(6):629-642.

**35.** Sherman KJ, Cherkin DC, Erro J, Miglioretti DL, Deyo RA. Comparing yoga, exercise, and a self-care book for chronic low back pain: a randomized, controlled trial. *Ann Intern Med.* Dec 20 2005;143(12):849-856.

**36.** Stulemeijer M, de Jong LW, Fiselier TJ, Hoogveld SW, Bleijenberg G. Cognitive behaviour therapy for adolescents with chronic fatigue syndrome: randomised controlled trial. *Bmj.* Jan 1 2005;330(7481):14.

**37.** Suter M, Giusti V, Worreth M, Heraief E, Calmes JM. Laparoscopic gastric banding: a prospective, randomized study comparing the Lapband and the SAGB: early results. *Ann Surg.* Jan 2005;241(1):55-62.

**38.** Tsauo JY, Leu WS, Chen YT, Yang RS. Effects on function and quality of life of postoperative home-based physical therapy for patients with hip fracture. *Arch Phys Med Rehabil.* Oct 2005;86(10):1953-1957.

**39.** Witt C, Brinkhaus B, Jena S, et al. Acupuncture in patients with osteoarthritis of the knee: a randomised trial. *Lancet.* Jul 9-15 2005;366(9480):136-143.
