# Supplementary material for: Development and Evaluation of a Pedagogical Tool to Improve Understanding of a Quality Checklist: A Randomised Controlled Trial
Source: PLoS Clin Trials. 2007 May 4;2(5):e22. doi: 10.1371/journal.pctr.0020022 (PMC1865084; doi:10.1371/journal.pctr.0020022)
Supplement: Text S5 — (102 KB DOC) [file pctr.0020022.sd008.doc]

**Text S5: description of the ICLS and panel of articles used for final assessment**

**ICLS**

Participants’ adherence to the ICLS training was very good. One participant did not complete the training because of a log-in problem. All other participants completed the entire training.

The intervention process is described in Table 1. The rate of correct answers for the first 2 passages proposed by the ICLS varied widely depending on the passage proposed to the participants. Some passages had more than two-thirds correct answers, whereas other passages had a low rate of good answers. For items related to participants’ adherence and to the follow-up schedule, the rate of correct answers was high for all passages proposed..

Qualitative assessment of the ICLS is presented in Table 2. Overall, participants were satisfied with the computer program. The duration of the training lasted from 30 min to 1 hour and was considered as an appropriate length for 75% of the participants. Most participants considered that the training was successful (85.7%) (meaning that they feel like they learned how to use CLEAR NPT but no details on that…), 66.7% answered that the interface was good or very good and 77.8% that the text was easily readable.

Characteristics of the panel of RCT articles used for final assessment

The characteristics of the panel of RCT articles are presented in Table 3.

These characteristics were the gold standard to which participants results were compared.

Table 1.A. Rate of correct answers for the first 2 passages for each item of the ICLS for each item of the CLEAR NPT

| Items | | **1^st^ Passage***  **N= 38 (%)** | **2^nd^ Passage***  **N =38 (%)** |
| --- | --- | --- | --- |
| item 1 | Was the generation of allocation sequences adequate? | 35 (92.1) | 22 (59.4) |
| item 2 | Was the treatment allocation concealed? | 4 (10.5) | 18 (48.6) |
| item 3 | Were details of the intervention administered to each group made available? | 30 (78.9) | 24 (63.2) |
| item 4 | Were care providers’ experience or skill in each arm appropriate? | 10 (26.0) | 24 (63.2) |
| item 5 | Was participant adherence assessed quantitatively ? | 35 (92.1) | 31 (81.6) |
| item 6 | Were participants adequately blinded? | 29 (76.3) | 33 (86.8) |
| item 7 | Were care providers or people caring for the participants adequately blinded? | 37 (97.4) | 24 (63.2) |
| item 8 | Were outcome assessors adequately blinded to assess the primary outcomes. | 4 (10.5) | 34 (91.9) |
| item 9 | Was the follow-up schedule the same in each group | 33 (86.8) | 34 (89.5) |
| item 10 | Were the main outcomes analyzed according to the intention-to-treat principle | 16 (42.1) | 35 (92.1) |

* First and Second Passage: All participants got to answer to the same item of CLEAR NPT twice with two different examples. Their first and second answers are called “1^st^ passage” and “2nd passage” in this table.

## Table 2 Qualitative analysis of the pedagological tool

|  | Answers  N= 36 | Answers  % |
| --- | --- | --- |
| Duration of the training |  |  |
| Less than 30 min | 5 | 13.9 |
| Between 30 min and 1 hour | 21 | 58.3 |
| More than 1 hour | 10 | 27.8 |
| Opinion of the duration of training |  |  |
| Too short | 0 | 0.0 |
| Long enough | 27 | 75.0 |
| Too long | 9 | 25.0 |
| **Opinion of the success of the training[[1]](#footnote-2)1** |  |  |
| Successful | 30 | 85.7 |
| Not successful | 5 | 14.3 |
| Quality of the Interface tool |  |  |
| Very bad | 0 | 0.0 |
| Bad | 2 | 5.5 |
| Average | 10 | 27.8 |
| Good | 19 | 52.8 |
| Very good | 5 | 13.9 |
| Quality of the text |  |  |
| Easily readable | 28 | 77.8 |
| Hardly readable | 8 | 22.2 |
| Not readable at all | 0 | 0.0 |

tABLE 3. Characteristics of the articles assessed according to the criterion standard

| Items | | N | Answers (%) | | | | |
| --- | --- | --- | --- | --- | --- | --- | --- |
|  | |  | **Yes** | **No** | **No because**  **blinding is**  **not feasible** | **No although**  **blinding is**  **not feasible** | **Unclear** |
| item 1 | Was the generation of allocation sequences adequate? | 39 | 61.5 | 0.0 | - | - | 38.5 |
| item 2 | Was the treatment allocation concealed? | 39 | 41.0 | 38.5 | - | - | 20.5 |
| item 3 | Were details of the intervention administered to each group made available? | 39 | 92.3 | 7.7 | - | - | 0.0 |
| item 4 | Were care providers’ experience or skill in each arm appropriate? | 39 | 51.3 | 0.0 |  |  | 48.7 |
| item 5 | Was participant (i.e., patients) adherence assessed quantitatively ? | 39 | 41.0 | 10.3 | - | - | 48.7 |
| item 6 | Were participants adequately blinded? | 39 | 20.5 | - | 74.4 | 5.1 | 0.0 |
| item 7 | Were care providers or people caring for the participants adequately blinded? | 39 | 2.5 | - | 94.9 | 2.6 | 0.0 |
| item 6.1 and 7.1 | If patients and / or care providers were not blinded, were all other treatments and care (i.e., co-interventions) the same in each randomized group? | 38 | 18.4 | 15.8 | - | - | 65.8 |
| item 6.2 and 7.2 | If patients and / or care providers were not blinded, were withdrawals and lost to follow-up the same in each randomized group? | 38 | 68.4 | 5.3 | - | - | 26.3 |
| item 8 | Were outcome assessors adequately blinded to assess the primary outcomes. | 39 | 46.1 | - | 33.3 | 20.5 | 0.0 |
| item 8.1 | If outcome assessors were not adequately blinded, were specific methods used to avoid ascertainment bias ? | 21 | 42.9 | 57.1 | - | - | 0.0 |
| item 9 | Was the follow-up schedule the same in each group | 39 | 89.7 | 2.6 | - | - | 7.7 |
| item 10 | Were the main outcomes analyzed according to the intention-to-treat principle | 39 | 46.1 | 46.1 | - | - | 7.7 |

1. ^1^ N = 35 [↑](#footnote-ref-2)
